# Development of a Novel Floating *In-situ* Gelling System for Stomach Specific Drug Delivery of the Narrow Absorption Window Drug Baclofen

**Published:** 2010

**Authors:** Rishad R. Jivani, Chhagan N. Patel, Dashrath M. Patel, Nurudin P. Jivani

**Affiliations:** a *Department of Pharmaceutics, Smt. R.B.Patel Mahila Pharmacy College, Atkot, Gujarat, India.*; b * Department of Pharmaceutical Analysis, Shari Sarvajanik Pharmacy College, Mehsana, Gujarat, India.*

**Keywords:** Narrow absorption window, Baclofen, Floating *in-situ *gel

## Abstract

The present study deals with development of a floating *in-situ *gel of the narrow absorption window drug baclofen. Sodium alginate-based *in-situ *gelling systems were prepared by dissolving various concentrations of sodium alginate in deionized water, to which varying concentrations of drug and calcium bicarbonate were added. Fourier transform infrared spectroscopy (FTIR) and differential scanning calorimetry (DSC) were used to check the presence of any interaction between the drug and the excipients. A 3^2^ full factorial design was used for optimization. The concentrations of sodium alginate (X_1_) and calcium bicarbonate (X_2_) were selected as the independent variables. The amount of the drug released after 1 h (Q_1_) and 10 h (Q_10_) and the viscosity of the solution were selected as the dependent variables. The gels were studied for their viscosity, *in-vitro *buoyancy and drug release*. *Contour plots were drawn for each dependent variable and check-point batches were prepared in order to get desirable release profiles. The drug release profiles were fitted into different kinetic models. The floating lag time and floating time found to be 2 min and 12 h respectively. A decreasing trend in drug release was observed with increasing concentrations of CaCO_3_. The computed values of Q_1_ and Q_10 _for the check-point batch were 25% and 86% respectively, compared to the experimental values of 27.1% and 88.34%. The similarity factor (*f*_2_) for the check-point batch being 80.25 showed that the two dissolution profiles were similar. The drug release from the *in-situ *gel follows the Higuchi model, which indicates a diffusion-controlled release. A stomach specific *in-situ *gel of baclofen could be prepared using floating mechanism to increase the residence time of the drug in stomach and thereby increase the absorption.

## Introduction

Alginic acid is a linear block polysaccharide copolymer made of β-D-mannuronic acid (M) and α-L-guluronic acid (G) residues joined by 1,4 glycosidic linkages. The proportion and the arrangement of the blocks along the polymer chain much depend on the algal source. The aqueous alginate solutions could form firm gels in presence of di- and tri-valent metal ions by a cooperative process involving consecutive guluronic residues in the G blocks of the alginate chain. This property has been widely used for preparation of vehicles for sustained delivery of the bioactive molecules (1-4).

Matrices containing sodium alginate and sodium-calcium alginate have been investigated for their sustained release effects (5-6). *In-vitro *release from capsules containing sodium alginate and calcium phosphate (7), and from solid beads containing a calcium alginate gel system have been reported (8-10). To achieve repeatability in gelation we used a source of Ca^++^ ions in the solution itself. Due to the free calcium ions being complexed with sodium citrate, gelation was delayed until the administered solution reached the acidic environment of the stomach. Gelation was then occurred as the complex broke down and the Ca^++^ ions were released. The optimum quantities of sodium citrate that maintained the fluidity of the formulation before administration and resulted in gelation after being added to simulated gastric fluid (pH = 1.2), was reported previously (11). The calcium carbonate present in the gelling formulation released carbon dioxide in gastric environment thereby making the formulation porous and buoyant and prolonging the residence time. This floating in stomach provides the potential to sustain the drug release over a long period of time. 

Some drugs show region-specific absorption that can be related to different solubility and stability in different regions of the intestine as a result of changes in environmental pH, degradation by enzymes present in the lumen of the intestine or interaction with endogenous compounds such as bile (12). Also, active drug transport mechanisms involving carriers and pump systems have been well described (13).

Baclofen has a narrow absorption window in small intestine thus showing a low bioavailability (14-15). It is difficult to formulate this drug into sustained release preparations because on arrival to colon (or even before) the absorption becomes low or nonexistent. Therefore, this study was aimed to increase the residence of baclofen at/ or above the absorption window by preparing a floating *in-situ *gelling system using sodium alginate (considering the fact that it is stable at gastric condition) by applying a 3^2 ^full factorial design (16). 

## Experimental

*Materials*

Baclofen was provided as a gift sample by Sun pharmaceutical Ltd., Vadodara, India. Sodium alginate was obtained as a gift sample from Shital Chemicals, Ahemdabad, India. Calcium bicarbonate was purchased from S.D. Fine Chemicals, Mumbai, India. All other ingredients, being of analytical grade, were obtained from Lesar chemicals, Vadodara, India. All materials used throughout the study conformed to the standards of USP 24.

*Methods*

*Fourier transform infrared spectroscopy (FTIR)*

The presence of any drug-polymer interaction was studied by FTIR spectroscopy. IR spectra for drug and the drug-loaded *in-situ *gels were recorded in a Fourier transform infrared (FTIR) spectrophotometer (FTIR-8400 S, Shimadzu, Japan) with KBr pellets. The scanning range was 400– 4000 cm^−1^.

*Differential scanning calorimetry (DSC)*

The DSC analysis of the pure drug and the drug-loaded *in-situ *gels was performed by using an automatic thermal analyzer system (*i.e. *DSC 60, Shimadzu, Japan) to evaluate the drug-polymer interactions (17). The analysis was performed at a rate of 20 ^o^C min from 50^ o^C to 300 ^o^C under a nitrogen flow of 25 mL/min. 

*Full factorial design*

Two factors each at three levels were selected and experimental trials were performed at all possible nine combinations. In the present investigation, the percentages of sodium alginate (X_1_) and calcium carbonate (X_2_) were chosen as the independent variables. The drug release after 1 h (Q_1_) and 10 h (Q_10_), and viscosity were selected as the dependent variables. The experimental design with the corresponding formulations is outlined in Table 2. Sodium alginate was used at 1.5%, 2%, and 2.5%, while calcium carbonate was used at 0.5%, 1% and 1.5%. A statistical model incorporating interactive and polynomial terms was used to evaluate the responses: Y=b_0_ +b_1_ X_1 _+b_2_ X_2 _+b_12_ X_1_X_2_ +b_11 _X_1_^2^ +b_22_ X_2_^2^, where Y is the dependent variable, b _0_ is the arithmetic mean response of the 9 runs and any *b*i is the estimated coefficients for the related factor Xi. The main effects (X_1_ and X_2_) represent the average result of changing one factor at a time from its low to high value. The interaction term “X_1_X_2_” shows how the response changes when the two factors change simultaneously. The polynomial terms (X_1_^2^ and X_2_^2^) are included to investigate nonlinearity.

*Preparation of the in-situ gelling solutions*

Sodium alginate solutions of different concentrations were prepared in deionized water containing 0.25% of sodium citrate. Low concentrations of cations in solution were sufficient to hold the molecular chains together and inhibit hydration. Sodium alginate solution was heated to 70 ^o^C with stirring. After cooling to below 40 ^o^C, different concentrations of calcium bicarbonate and the drug were added and dispersed well with continuous stirring. The resulting sodium alginate *in-situ *gelling solution containing baclofen was finally stored in amber bottles until further use.

*Viscosity measurement of the in-situ gelling solutions*

Viscosity of the sols were determined using a Brookfield digital viscometer (Model no LVDV 2P230) with the spindle number 1. The temperature of the 2 mL samples was kept at 25 ±1 °C during each measurement which lasted 30 sec, and the experiments were performed in triplicate.

*In-vitro buoyancy*

The *in-vitro *buoyancy study was performed using the USP dissolution apparatus II with 500 mL of simulated gastric fluid (pH = 1.2). The medium temperature was kept at 37 ^o^C. A 10 mL sample of the prepared solution (*in-situ *gelling formulation) was drawn up with the help of a disposable syringe and placed into a Petri dish. Then, the Petri dish was placed in the dissolution vessel containing the medium without much turbulence. The time for the gel to come to surface (floating lag time) and the time the gel remained floated on the medium surface (floating time) were recorded (18).

*In-vitro drug release*

The study of the baclofen release from the *in-situ *gelling preparation was carried out similar to the method described by Zatz and Woodford (19) with some modification using USP 24 dissolution test apparatus with paddle stirrer at a rate of 50 rpm. The slow speed prevented breaking of the gelled formulation and ensured a low level of agitation. The dissolution medium used was 500 mL of a 0.1 N solution of HCl (pH = 1.2), and the temperature was kept at 37 ^o^C. A 10 mL sample was withdrawn using a disposable syringe; the needle was then wiped clean and the excess sample removed from the needle end. The sample was then gently transferred into a Petri dish which was then immersed into the dissolution medium without much turbulence. At 1 h intervals, an accurately measured sample of the dissolution medium was removed for analysis and replaced with the same amount of the pre-warmed (37 ^o^C) fresh medium. The absorbance of the sample was measured at 267 nm using a UV spectrophotometer (Shimadzu, UV-1601, Japan) for analysis of baclofen. Each experiment was performed for a period of 12 h in triplicate.

*Analysis of the drug release data*

The method of Bamba *et al. *was adopted to study the kinetics of drug release (20). The *in- vitro *drug release data were analyzed by fitting them into different kinetic models in order to investigate the release mechanism of baclofen from the gel systems (21-26). The least values for the sum of the square of residuals (SSR) and Fisher’s ratio (F) were used to select the most appropriate kinetic model.

*Statistical analysis*

The statistical analysis of the factorial design batches was performed by multiple regression analysis using Microsoft Excel^®^. To evaluate the contribution of each factor to the effects on the responses, two-way analysis of variance (ANOVA) followed by Tukey test was performed using the Sigma State software (Sigma State 3.5. SPSS, USA). To graphically show the influence of each factor on responses, the contour plots were generated using the Sigma Plot Software (Sigma Plot Software 11.0 SPPS, USA). The significance level was considered to be p < 0.05. 

*Criteria for the optimized batch*

Three limits were arbitrarily selected based on the theoretical release profile calculated by the pharmacokinetic parameters of balcofen: (1) Percentage of drug release after 1 h (Q_1_) = 25% (2) Percentage of drug release after 10 h (Q_10_) = 86%, and (3) Viscosity of the solution = 210-250cps.

*Comparison of the dissolution profiles*

The similarity factor (*f*_2_) given by SUPAC guidelines for modified release dosage forms was used as a basis to compare the dissolution profiles. The dissolution profiles were considered similar when *f*_2_ was in the range of 50 to 100. The similarity factor was calculated by the following formula:

f2=50 log1+1n∑t=1n(Rt-Tt)2-0.5×100

Where n is the number of the sampling time, and R_t_ and T_t_ are the reference and test dissolution values at time t, respectively.

## Results and Disscusion

**Figure 1:**
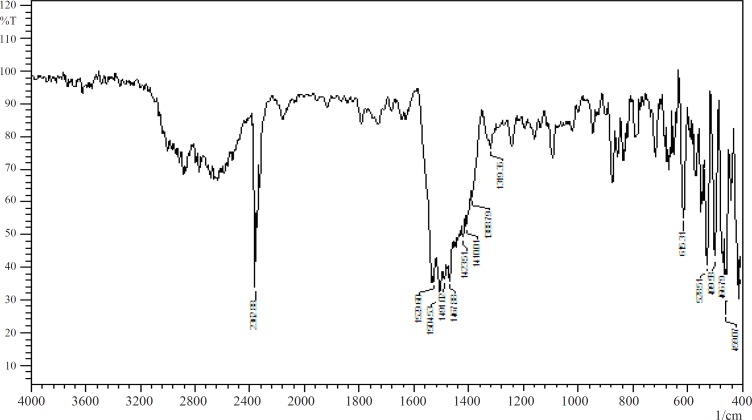
FTIR spectrum of baclofen

**Figure 2:**
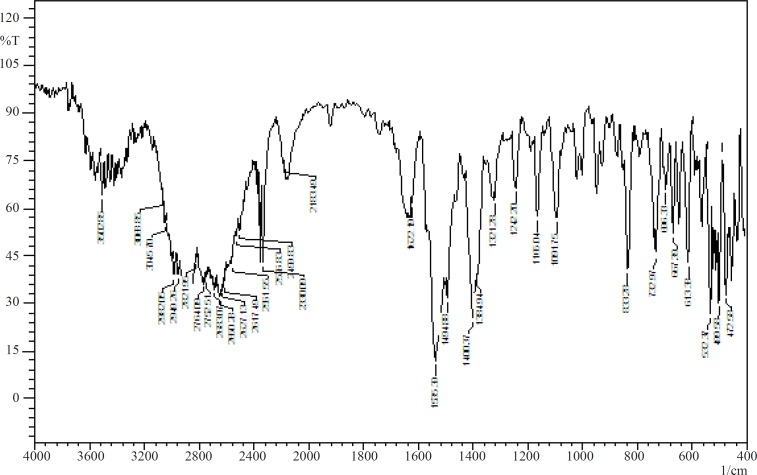
FTIR spectrum of *in-situ *gelling formulation

The stability of the drug and sodium alginate in presence of each other was studied by IR spectroscopy and DSC. Figure 1 and Figure 2 show the IR spectra of pure baclofen and the combination formulation. Major peaks assigned to baclofen due to its functional groups (-C-Cl, -COOH, and -NH_2_) are 1100, 1530, and 1610cm^1^ (Figure 1). These characteristic peaks were also observed in the combination formulation (Figure 2), which indicated that no major interaction occurred between the functional groups of baclofen with the other ingredients. 

**Figure 3:**
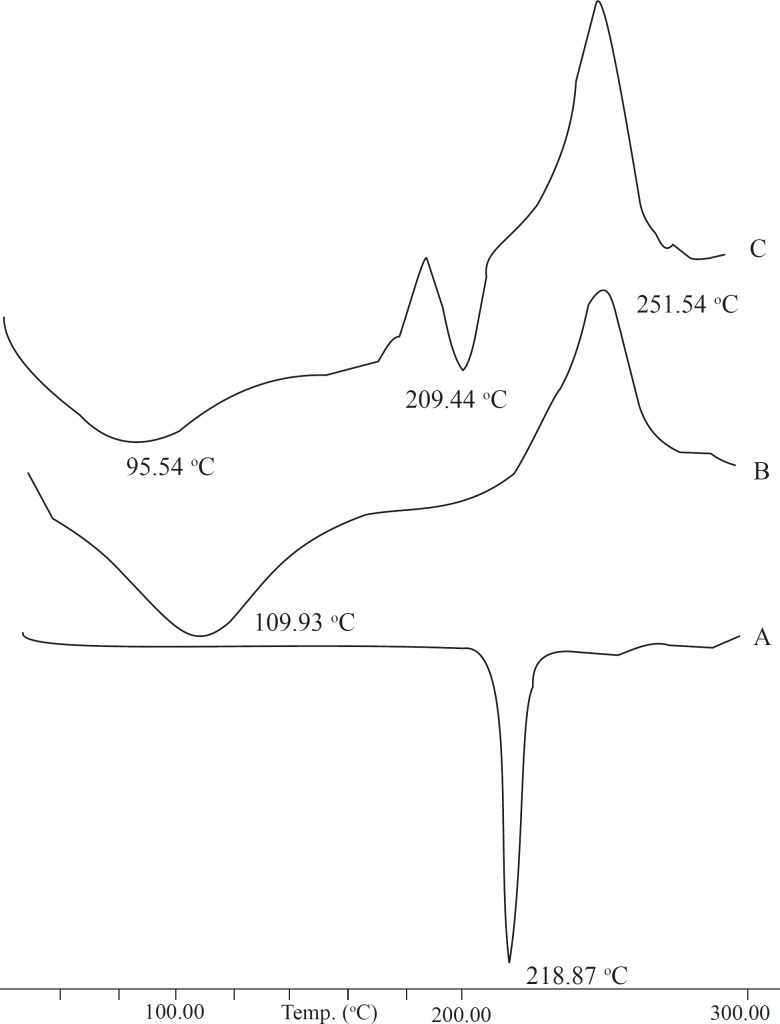
DSC thermographs: (A) Pure baclofen (B) Sodium alginate (C) Drug-loaded formulations.

The DSC results provide both qualitative and quantitative information about the physicochemical state of the drug present in formulation. The DSC thermograph of the pure drug, sodium alginate, and combination formulation were obtained (Figure 3). The thermograph of pure baclofen showed a melting endothermic peak at 218.87 ^o^C. The thermograph of the drug-loaded formulation showed two different peaks related to baclofen (209.44 ^o^C) and sodium alginate (95.54 ^o^C). This confirmed that the presence of other excipients did not affect the drug stability.

All prepared batches of the factorial design were evaluated for their floating properties in simulated gastric fluid. The time for formulation to come to the medium surface (floating lag time) and the time the formulation maintained floated on the medium surface (duration of floating) were determined (Table 1). 

**Table 1:** *In-vitro *buoyancy of baclofen formulations

**Formulation**	**F**_1_	**F**_2_	**F**_3_	**F**_4_	**F**_5_	**F**_6_	**F**_7_	**F**_8_	**F**_9_
**Floating lag time (sec)**	80	60	50	80	60	50	80	60	50
**Floating time (h)**	12	12	12	12	12	12	12	12	12

Upon contact with an acidic medium, calcium carbonate effervesced, releasing carbon dioxide and calcium ions. Then, gelation and cross-linking by Ca^++^ ions took place to provide a gel barrier at the surface of the formulation. The released carbon dioxide was entrapped in the gel network producing a buoyant preparation, which resulted in extended floating. Then, the calcium ion reacted with sodium alginate to produce a cross-linked three-dimensional gel network that may restrict further liberation of carbon dioxide and drug molecules, which resulted in an extended period of drug release (28-29). The floating properties of the formulation mainly depend on calcium carbonate and sodium alginate concentrations. The lowest amount of calcium carbonate which produced a buoyant gel system for the duration of drug release study was found to be 0.5% at all polymer levels. On increasing the calcium carbonate concentration, the floating lag time was reduced and the duration of floating was extended. The increasing amounts of Ca^++ ^and CO_2_ resulted from the increase in calcium carbonate concentration, are responsible for the observed reduction in floating lag time and increasing duration of floating. Similarly an increase in polymer concentration resulted in decreased floating lag time and increased floating duration of the prepared systems (30).

The percent drug release after 1 h (Q_1_) and 10 h (Q_10_), and the viscosity results showed wide variation (Table 2). From the results of multiple regression analysis, it was found that both factors had statistically significant influence on all dependent variables (p < 0.05, Table 3). The high values of coefficients calculated from the multiple regression analysis clearly indicate that the responses were strongly dependent on the factors studied (Table 3). 

**Table 2:** Design layout for “3^2^” factorial design

**Batch code**	**Real values**		**Transformed values**		**Dependent variables**		
	**X**_1_	**X**_2_	**X**_1_	**X**_2_	**Q**_1_	**Q**_10_	**Viscosity**
**F1**	1.5%	0.5%	-1	-1	50.9 ± 1.46	99.89 ± 2.12	112 ± 8.76
**F2**	1.5%	1%	-1	0	55.45 ± 2.28	99.08 ± 1.45	134 ± 4.74
**F3**	1.5%	1.5%	-1	1	39.5 ± 2.16	93.98 ± 2.77	154 ± 8.11
**F4**	2%	0.5%	0	-1	34.5 ± 2.41	91.81 ± 3.49	225 ± 10.73
**F5**	2%	1%	0	0	27.7 ± 1.78	87.02 ± 4.82	236 ± 7.90
**F6**	2%	1.5%	0	1	33.15 ± 2.09	85.29 ± 2.66	266 ± 5.53
**F7**	2.5%	0.5%	1	-1	25.9 ± 1.88	77.87 ± 2.83	295 ± 8.29
**F8**	2.5%	1%	1	0	20 ± 0.07	73.91 ± 3.65	330 ± 11.67
**F9**	2.5%	1.5%	1	1	14.5 ± 0.96	63.19 ± 2.00	359 ± 14.44

X_1_ is the percentage of sodium alginate, X_2_ is the percentage of calcium carbonate, Q_1_ is the percent drug release during the first hour, and Q_10_ is the percent drug release after 10 h. Viscosity is shown in cps (n = 3). All batches contain 20 mg of baclofen

**Table 3:** Summary of regression output of significant factors for measured responses

**Responses**	**Co-efficients of parameters**						
	**b**_0_	**b**_1_	**b**_2_	**b**_22_	**b**_12_	**b**_22_	R^2^
**Q**_1_	32.65	-4.02	-14.24	-1.308	2.59	-1.77	0.914
**Q**_10_	88.92	-4.51	-12.99	-1.33	-3.58	-2.913	0.989
**Viscosity**	241.1	24.5	97.33	1.83	-11.66	5.5	0.998

For graphical representation of the influence of factors, the contour plots were generated for all dependent variables. To evaluate the relative contribution of the different levels of each factor, two-way ANOVA was performed followed by Tucky test, and the results were depicted in Table 4. 

**Table 4:** Results of the two-way ANOVA for dependent variables

**Source of variation**	**DF**	**SS**	**MS**	**F**		**P**
		**Q**_1_				
**% of Sodium alginate**	2	100.6	50.3	1.6		0.308
**% of Calcium carbonate**	2	1230.3	615.1	19.63		0.009
**Residual**	4	125.32	31.3			
**Total**	8	1456.33	182.0			
		**Q**_10_				
**% of Sodium alginate**	2	126	63.0	7.714		0.04
**% of Calcium carbonate**	2	1036.4	518.2	63.43		0.001
**Residual**	4	32.67	8.1			
**Total**	8	1195.13	149.3			
		**Viscosity**				
**% of Sodium alginate**	2	3608	1804	31.49		0.004
**% of Calcium carbonate**	2	57114	28857	498.57		0.001
**Residual**	4	229	57.27			
**Total**	8	60952	7619			

Q_1_ is the percent drug release during the first hour, and Q_10_ is the percent drug release after 10 h. Viscosity is measured in cps. DF is the degree of freedom, SS is the sum of squares and F is the Fischer’s ratio.

**Figure 4:**
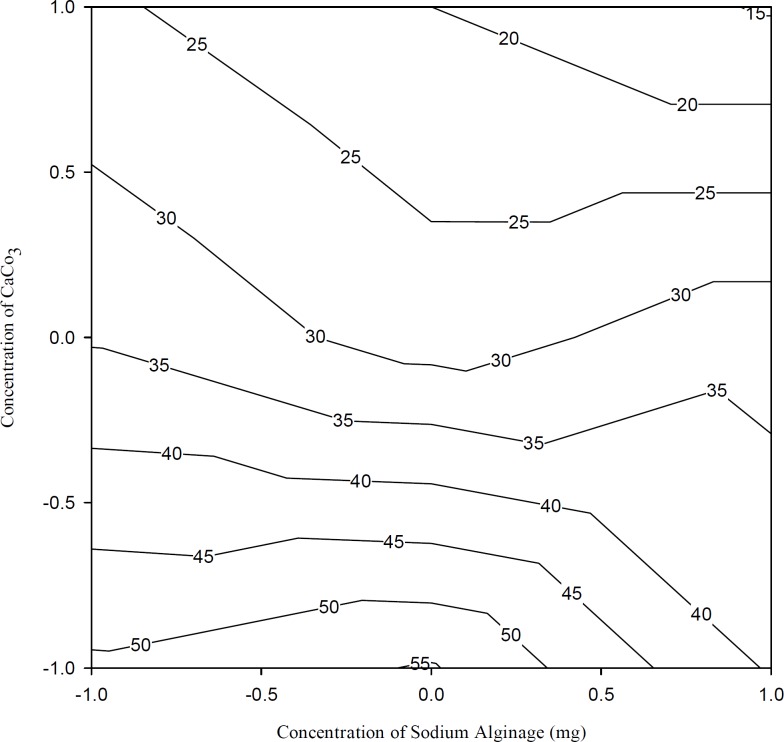
Contour plots showing the drug release during the first hour (Q_1_) at different combinations of X_1_ and X_2_. The contour lines show the percent drug release at the end of the first hour

Figure 4 shows the drug release after 1 h. From the results of the multiple regression analysis and two-way ANOVA, it was noticed that the percentage of calcium carbonate had a significant influence on the release rate at the first hour (p < 0.05, Table 3 and Table 4). Initially, the drug was released at a faster rate due to the burst effect. As the percentage of calcium carbonate increased, the release rate decreased due to the stronger gel formation occurred in presence of increasing concentrations of Ca^++^ ions, which leads to a slower release of the drug from these gels.

**Figure 5:**
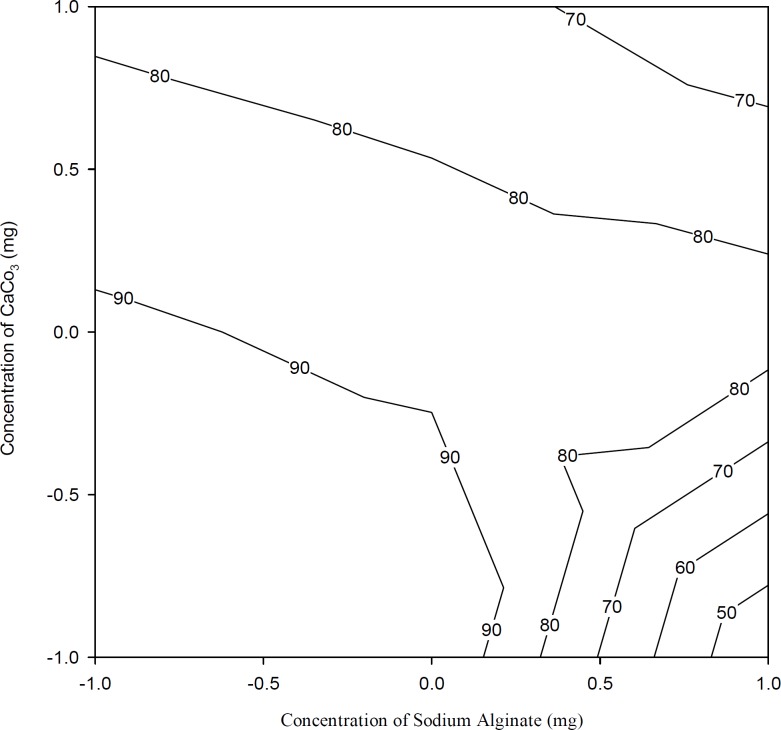
Contour plots showing the drug release after 10 h (Q_10_) at different combinations of X_1_ and X_2_. The contour lines show the percent drug release at the end of the 10th hour

Figure 5 shows the drug release after 10 h. From the results of the multiple regression analysis and two-way ANOVA, it was observed that both factors, *i.e. *the percentages of sodium alginate and calcium carbonate, had significant influence on the release rate after 10 h (p < 0.05, Table 3 and Table 4). A significant decrease in the rate and extent of drug release was observed with the increase in polymer concentration in *in-situ *gelling preparations. These may be attributed to the increase in density of the polymer matrix and also an increase in the diffusion path length which the drug molecules have to pass. 

The drug release from the gel was characterized by an initial phase of high release (burst effect). However, as gelation proceeded the remaining drug was released at a slower rate comprising the second phase of release, *i.e. *a moderate release rate. This bi-phasic pattern of release is a characteristic of the matrix diffusion kinetics (27). The initial burst effect was considerably reduced with an increase in polymer concentration. 

**Figure 6:**
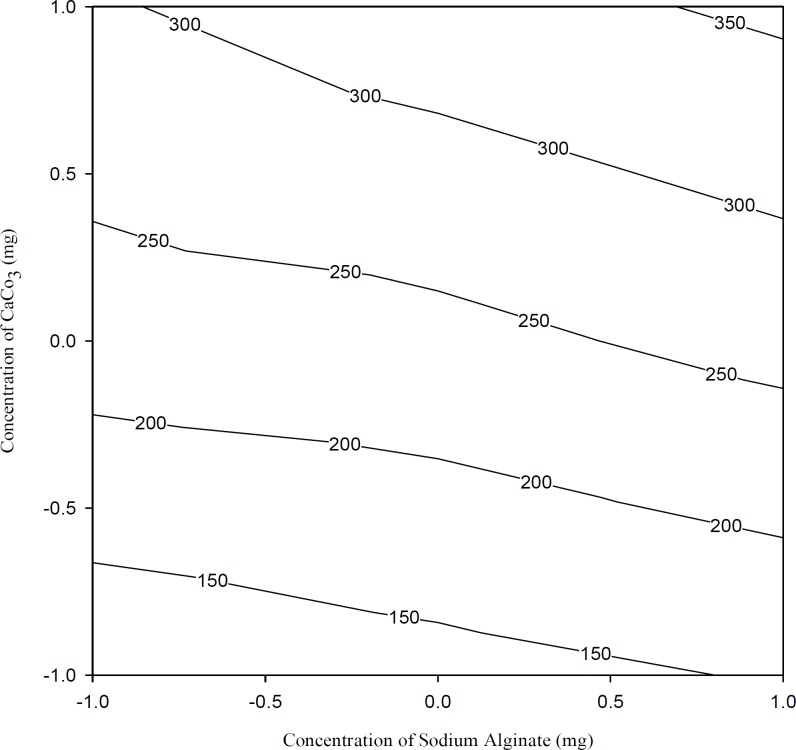
Contour plots showing viscosity at different combinations of X_1_ and X_2_. The contour lines show the viscosity

Figure 6 shows the effects of the polymer and calcium carbonate concentrations on viscosity of the solutions. It was observed that calcium carbonate concentration had a significant effect on viscosity (p < 0.05, Table 3 and Table 4). Increasing calcium carbonate concentration in formulations increased the viscosity at all polymer concentrations studied. 

Since calcium carbonate in formulations was present in the form of insoluble dispersion, an increase in its concentration proportionally increased the number of particles dispersed, thus contributing to the increased viscosity. In view of their oral administration, the rheological properties of the solutions are of importance. In selection of the gelling polymer concentration, a compromise is sought between a sufficiently high concentration for formation of gels of satisfactory gel strengths for the desirable release rate, and a sufficiently low concentration to maintain an acceptable viscosity for ease of swallowing.

**Figure 7:**
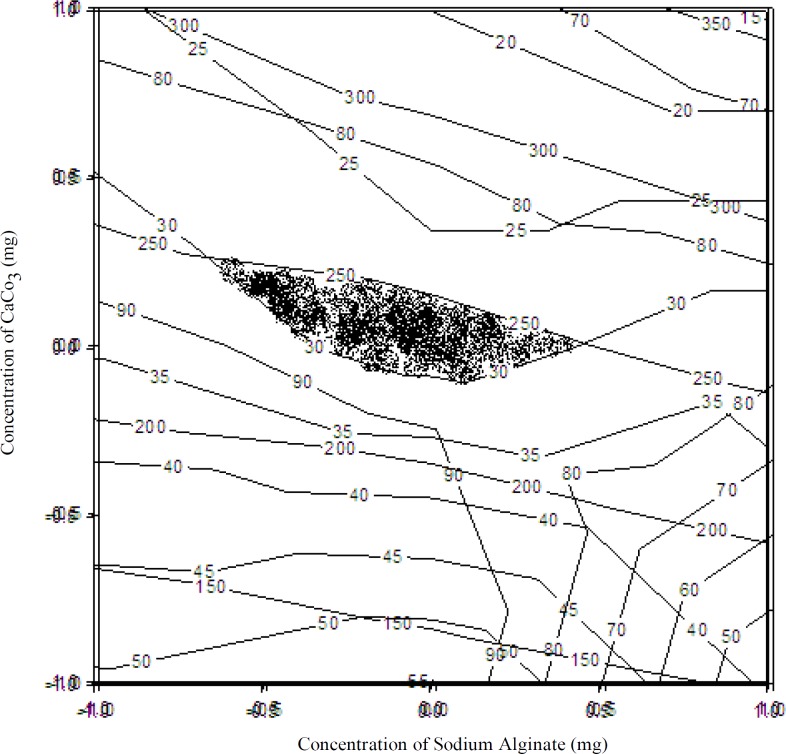
Overlapping region of the contour plots of all variables

Figure 7 shows overlapping region of the contour plots of all dependent variables. To find out the optimized area, overlapping region of all three contour plots was shown. The highlighted area was the optimized area, and a check-point batch was prepared considering X_1_= 2.0% and X_2_= 1.2%.

Similarity factor (*f*_2_) was calculated considering the ideal release profile as the reference and the check-point batch as the test formulation. The value of *f*_2_ was found to be 80.02, which revealed that the two dissolution profiles were similar.

**Figure 8:**
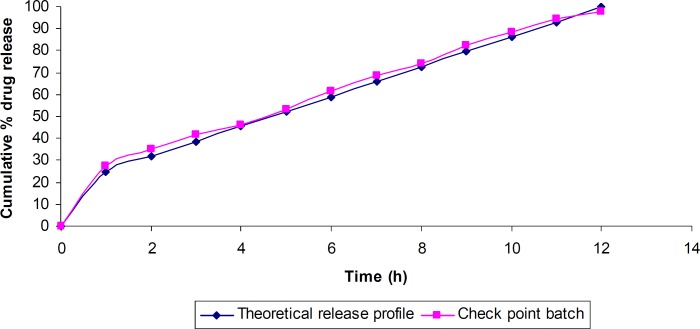
Theoretical and the check-point batch release profile

The predicted (theoretical) and observed dissolution profiles for the check-point batch was depicted in Figure 8. The computed values for Q_1_ and Q_10_ for the check-point batch were 25% and 86% respectively, while the experimental values were 27.1% and 88.34% respectively. 

The *in-vitro *drug release data of the check-point batch were analyzed for determining the kinetics of drug release. The zero-order, first-order, Higuchi, Hixson-Crowell, Korsmeyer-Peppas and Weibull models were tested. The Higuchi model showed the least sum of the squares of residuals (SSR = 75.8) and the Fischer’s ratio (F = 14.8). The release of baclofen from the *in-situ *gel followed higuchi model and the release process was found to occur by an anamolous diffusion-controlled mechanism.

## Conclusion

This study showed the feasibility of *in-vitro *gel forming from aqueous solutions of sodium alginate containing Ca^++^ ions in a complexed form. Furthermore, it was observed that the resulting gel remained buoyant for 12 h and slowly released balcofen during the 12 h period. It is concluded that baclofen could be targeted to stomach and be released slowly over a long period of time. 
